# Provider experiences with and attitudes about an embedded pragmatic clinical trial

**DOI:** 10.1186/s12875-025-02799-w

**Published:** 2025-04-23

**Authors:** Sarah M. Leatherman, Britte Beaudette-Zlatanova, Gregory Robben, Peter A. Glassman, Patricia Woods, Ryan E. Ferguson, William C. Cushman, Areef Ishani

**Affiliations:** 1 Cooperative Studies Program Coordinating Center, VA Boston Healthcare System, Boston, MA USA; 2 Department of Biostatistics, Boston University School of Public Health, Boston, MA USA; 3 Pharmacy Benefits Management Services, Department of Veterans Affairs, Washington, DC USA; 4 VA Greater Los Angeles Healthcare System, Los Angeles, CA USA; 5 David Geffen School of Medicine at UCLA, Los Angeles, CA USA; 6 Department of Medicine, Boston University School of Medicine, Boston, MA USA; 7 Medical Service, Memphis VA Medical Center, Memphis, TN USA; 8 Department of Preventive Medicine, University of Tennessee Health Science Center, Memphis, TN USA; 9 Minneapolis VA Healthcare System, Minneapolis, MN USA; 10 Department of Medicine, University of Minnesota, Minneapolis, MN USA

**Keywords:** Primary care, Pragmatic clinical trials, Provider experience, Embedded trials, Hypertension, Diuretics

## Abstract

**Background/aims:**

The Diuretic Comparison Project (DCP) was a pragmatic clinical trial comparing rates of cardiovascular events between hydrochlorothiazide or chlorthalidone. VA primary care providers (PCPs) and their patients were participants in the study. Veterans ≥ 65 years taking hydrochlorothiazide were randomized to continue on hydrochlorothiazide or switch to chlorthalidone. Participating providers could decline the randomization of their patients. Providers were surveyed about their experience with DCP, and to ascertain providers’ understanding of and attitudes towards embedded pragmatic trials.

**Methods:**

A questionnaire was emailed to PCPs that provided informed consent to participate in the study. The survey asked about provider experience with the trial including interest in the study question, awareness of the study and educational materials, impact on the provider-patient relationship, burden of study participation, and their attitudes towards pragmatic trials. Respondents could also add free text comments.

**Results:**

There were 180 completed surveys. Of those, most found the trial question of interest (91%) and found the time required to participate in the trial was reasonable (67%). Only 2 (1%) felt the study had a negative impact on the provider-patient relationship. 97% of providers were as comfortable with (59%) or more comfortable with (32%) DCP compared to traditional randomized controlled trials.

**Conclusion:**

Responding providers’ experience with DCP and their attitudes towards pragmatic trials were positive. Primary care providers indicated willingness to participate in future pragmatic trials if burden is low and it does not negatively impact patient care. Results support continued use of pragmatic embedded clinical trials in primary care.

**Clinical trial registration:**

NCT02185417. Registered 9 July 2014. https://clinicaltrials.gov/ct2/show/NCT02185417.

## Background

Point of Care (POC) Research is a novel pragmatic approach to clinical study design that embeds trials, to the extent possible, into usual clinical care [1–4]. It is uniquely positioned to compare the safety or efficacy of two or more approved treatments or diagnostic techniques under real-world circumstances. These trials take advantage of the electronic health record (EHR) to facilitate participant recruitment and follow-up, minimizing study burden and streamlining the experience for providers.

The Diuretic Comparison Project (DCP) was the first large-scale POC clinical trial conducted within the Veterans Affairs (VA) Healthcare System [5, 6]. The goal of the DCP study was to compare the effectiveness of two commonly used, FDA-approved medications in hypertensive patients while minimizing disruption to the usual care ecosystem. Patients were followed for a median of 2.4 years, when the target number of primary outcome events was reached, for major cardiovascular events and non-cancer deaths [7]. 69% of primary care providers approached, consented to participate in the study. The trial enrolled more than 4,000 providers and over 13,500 of their respective patients at 72 VA healthcare systems [6, 7]. Providers were included as participants in DCP to allow the investigators to learn more about their prescribing and care habits, the impact of the trial on patient-provider relationships, and to query providers about their experience with the trial.

The impact of traditional clinical trials on clinical care [8], patient relationships [9–11], and provider workload is well-documented in the literature. However, there is limited understanding on provider acceptance of pragmatic trials. We know that early and continued engagement of both the healthcare system and providers is critical to the success of such studies [12]. Previously identified provider concerns with clinical trials include: (1) increased clinical burden, (2) validity and reliability of results, and (3) the provider-patient relationship [13–15]. These concerns influenced the design of the DCP. Providers did not need to identify or consent eligible patients, and providers were not required to conduct any visits or lab tests that were not part of usual care for consented patients. Providers and patients at small, rural VA medical centers without research infrastructure were able to participate due to recruitment, randomization, and study data collection being handled off-site thereby enhancing the generalizability of the study findings. To respect the autonomy of both the provider and the patient, providers had to provide informed consent to participate in the study for their patients to be eligible and then patients of consented providers had to provide informed consent for themselves to participate in the study. In addition, providers had the ability to decline the randomization of any of their individual patients if they felt it would not be in the best interest of the patient.

Given the novelty of this study design, the paucity of data on provider participation in embedded POC trials, and the influence of previous focus groups on the design of current and future studies, the impact on providers and their relationship with their patients is of high interest. As a component of the trial, a questionnaire related to experiences with DCP and POC study designs in general was sent to providers consented during the course of DCP. The results of that survey and a discussion of the potential impact of an embedded clinical trial on providers and future clinical trial conduct are detailed below.

## Methods

Design details of DCP and full results of the trial have been previously described [5–7]. Briefly, the study was fully decentralized with all recruitment, randomization, passive follow-up and data collection executed by research staff at the Boston and Minneapolis VA medical centers. Primary care providers at every facility were offered educational materials about the trial prior to study launch through email, mail, staff meeting presentations, and the web. The trial recruited between June 2016 and November 2021 and completed follow-up in June 2022.

After receiving the educational materials about the trial, providers had the opportunity to consent to participate in the study, or decline participation, through the computerized patient record system (CPRS), the VA’s EHR. If providers consented to participate, their eligible patients were mailed information about the study and then contacted by phone to provide informed consent or decline participation in the study. Once a provider’s patient consented to participate in the study, the provider was sent an order in CPRS requesting the provider to approve or decline randomization. The trial was fully unblinded (i.e. both patients and providers were aware of the randomization assignment). If the provider declined randomization of the patient, the patient was informed. If the provider approved, the provider was sent a randomized drug order to sign, the patient was informed of their drug assignment and received their randomized medication by mail. Providers were expected to offer usual clinical care to their randomized patients, including management of study medications and follow-up of any adverse events. Providers could change the dose or discontinue the assigned drug for a patient at any time. The average number of patients randomized per provider was 4 (ranging from 0 to 33).

In August 2022, after trial completion, consented providers of enrolled patients were emailed an IRB-approved survey using Microsoft Forms to assess experiences with and attitudes about DCP and POC trials. The survey questions were based on the results from a focus group study that explored provider perceptions of POC research and identified concerns that providers had with this type of research [13] as well as provider feedback that was received throughout the course of the study. The survey was reviewed by the VA Organizational Assessment Committee and the union notified. The survey was open to response for 4 weeks, 2 reminder emails were sent, and no incentives were offered for completion. Survey responses were anonymous. Providers that had left the VA since agreeing to participate or who no longer had a valid VA email address were excluded from the survey. Consent to participate in DCP and completion of the survey indicated consent to the survey.

**Fig. 1:**
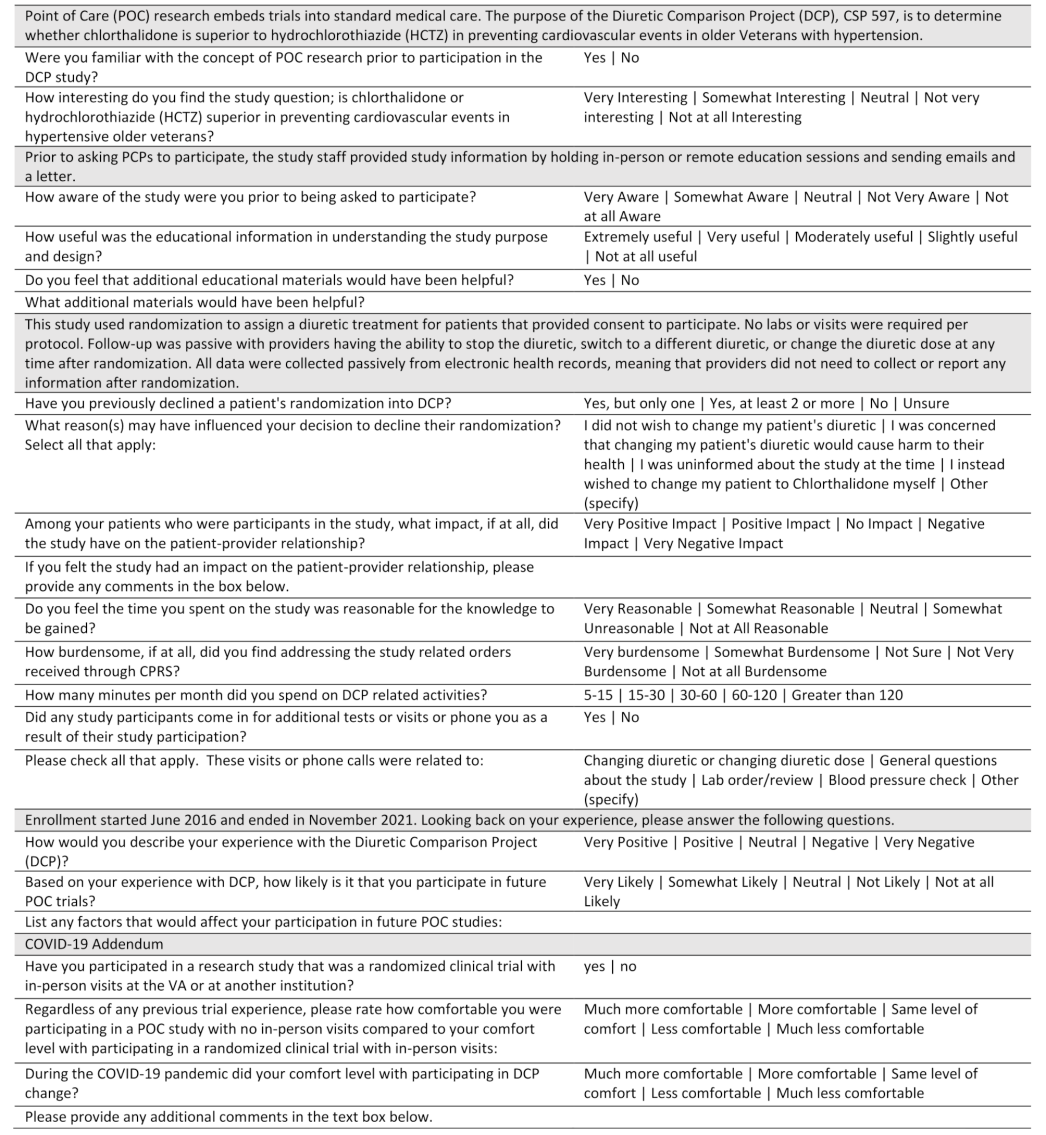
Provider experience with DCP survey

The survey queried providers about their familiarity with POC research and DCP, the impact of DCP on their clinical activities including patient relationships and time required, and their impressions of DCP and POC studies (Fig. 1). The survey allowed for open-ended comments throughout and for any additional commentary at the end of the questionnaire. Providers were also queried on basic demographic information including age and clinical experience, though geographic location was not solicited.

Survey results were summarized with frequencies and relative frequencies. Open-ended comments were categorized into themes identified post hoc.

## Results

The DCP enrolled primary care providers at over 500 VA outpatient facilities in all 50 states and Puerto Rico [6, 7]. Of the 4,128 providers enrolled, 180 (4%) providers completed the survey. Respondents were mostly experienced clinicians with at least 50% of their workload at the VA (Table 1). Respondents were similar to all consented DCP providers with respect to age and time as a VA clinician.

Most respondents were aware of both POC and DCP prior to participation in the trial (64% and 51%, respectively) and 91% found the study question of DCP of interest (Table 2). Only 1% of respondents reported that DCP had a negative impact on their patient relationships. Less than 5% reported that the time required for participation was burdensome or unreasonable. Two respondents indicated that they would not be interested in participating in future POC trials.

Of respondents, 19% wanted additional educational material, with 9% saying they did not receive the materials sent by the study team and 3% explicitly stating they would like follow-up data or study updates (Table 3).

Only 5% of respondents reported declining patient randomization, with 70% of those expressing concern about changing their patient’s diuretic. Overall, providers declined randomization for 7% of consented patients [6]. 

4% of respondents found addressing the study orders via the EHR to be burdensome and 1% thought the time they spent on the study was unreasonable for the potential knowledge to be gained. The majority of providers (84%) reported spending 15 min or less on DCP related activities each month.

Of the 180 respondents, 24 (13%) indicated scheduling additional visits as a result of the trial. Reasons for these visits included lab orders and reviews, blood pressure checks, and diuretic changes or dose changes.

While 17% of respondents reported a positive impact of the study on their relationship with patients, 1% indicated the study had a negative impact.

Zero respondents reported a negative experience with the trial. Many providers indicated willingness to participate in future POC trials, citing study simplicity and general enthusiasm for research as reasons for continued participation. 97% of providers were more or much more comfortable with DCP compared to traditional randomized clinical trials. Additionally, several respondents reported through open comments a willingness to participate in the future if there was additional education for providers and more study updates.

**Table 1:** Provider characteristics

Demographic characteristic	Consented Providers *N* = 4,128	Survey Respondents *N* = 180
Age*		
< 30 30–39 40–49 50–59 60–69 70–79 > 79	13 (0.4%) 343 (9.7%) 759 (21.5%) 1,131 (32.0%) 1,040 (29.4%) 230 (6.5%) 21 (0.6%)	-- 25 (13.9%) 45 (30.0%) 58 (32.2%) 44 (24.4%) 5 (2.8%) --
Years of experience		
< 5 5–10 > 10	-- -- --	7 (3.9%) 30 (16.7%) 140 (77.8%)
Years at VA**		
< 5 5–10 > 10	1,004 (26.5%) 1,079 (28.5%) 1,702 (45.0%)	38 (21.1%) 60 (33.3%) 79 (43.9%)
VA appointment***		
≤ 50% 50–88% >88%	-- -- --	45 (25.0%) 70 (38.9%) 65 (36.1%)

*591 consented providers were missing age

**3,785 consented providers had VA time in service information available

***VA appointments are calculated in eighths

**Table 2:** DCP provider survey results

Survey question	Survey Respondents *N* = 180
Provider was familiar with POC trials prior to participation	116 (64.4%)
Provider interest in study question	
Somewhat or very interesting Neutral Not very or not at all interesting	163 (90.5%) 16 (8.9%) 1 (0.6%)
Awareness of study prior to invitation	
Somewhat or very aware Neutral Not very or not at all aware	89 (49.4%) 16 (8.9%) 80 (44.4%)
Usefulness of educational material	
Very or extremely useful Moderately or slightly useful Not at all useful	93 (51.7%) 76 (42.2%) 9 (5.0%)
Additional education materials would have been helpful	33 (18.3%)
Declined a patient’s randomization into DCP	10 (5.6%)
Reason for declining randomization^1^	
Did not wish to change patient’s diuretic Concerned changing diuretic would cause harm to patient Wish to changed patient’s diuretic myself Other	2 (20.0%) 4 (40.0%) 1 (10.0%) 3 (30.0%)
Impact of DCP on the patient-provider relationship	
Positive or very positive impact No impact Negative or very negative impact	28 (15.6%) 149 (82.8%) 2 (1.1%)
Time spent on the study was reasonable	
Somewhat or very reasonable Neutral Somewhat or very unreasonable	120 (66.7%) 53 (30.1%) 2 (1.1%)
Burden of addressing study-related orders	
Somewhat or very burdensome Not Sure Not very or at all burdensome	7 (3.9%) 24 (13.3%) 148 (82.2%)
Time spent per month on DCP activities	
0–15 min 15–30 min More than 30 min	151 (83.9%) 20 (11.1%) 6 (3.3%)
DCP participants came in for additional tests or visits	24 (13.3%)
Describe your experience with DCP	
Positive or very positive Neutral Negative or very negative	116 (64.4%) 64 (35.6%) 0
Likelihood of participating in another POC trial	
Somewhat or very likely Neutral Not or not at all likely	158 (87.8%) 19 (10.6%) 3 (1.7%)
Prior experience with traditional clinical trials	73 (40.6%)
Comfort level with DCP compared to traditional RCTs	
More or much more comfortable Same level of comfort Less or much less comfortable	67 (37.2%) 107 (59.4%) 5 (2.8%)
Comfort level with DCP changed during the COVID-19 pandemic	
More or much more comfortable Same level of comfort Less or much less comfortable	10 (5.6%) 161 (90.4%) 7 (3.9%)

^1^ Percentages calculated of those that reported declining randomization

**Table 3:** Factors and opinions impacting provider participation in point of care studies

Theme	Description	Example Survey Comments
Time	Providers are busy with non-research duties and are more willing to participate in research when time commitments are reasonable.	“This was nice that the provider had minimal involvement and the work was mostly done by the study and burden not placed on primary care or the providers.” “I am a busy clinician. Having a pragmatic RCT that minimizes the burden on the PCP is important in this day and age.” “Requirement for extra visits would discourage me.” “Honestly while I remember being told about the study and was happy to participate I do not remember spending any time on the study or it having any impact on care which I guess is a good thing” “This was nice that the provider had minimal involvement and the work was mostly done by the study and burden not placed on primary care or the providers” “Excellent job. Happy to participate. Minimal to no time required by PCPs is the key. There are always a few pt questions and some want to talk to their PCP: that’s true of any study. Fine. After that: PCP workload mgmt is the critical factor in success of these studies.”
Education	Providers want to know about research taking place with their patients, including updates during the study where possible.	“I didn’t receive any education material our have any interaction with DCP outside of the email asking for participation.” “Ongoing updates on enrollment (or pauses due to challenges)” “It would be nice if you sent an abstract summarizing the study’s results directly to clinicians who participated in the study. You know our emails and watching journals may not be everyone’s habits.” “I think the educational information prior to the study was critical.” “You could/should have sent prescriber participants an annual ‘POC research update’.”
Safety	Providers primary priority with their patients is safety.	“Baseline safety of the meds being studied [would affect my decision to participate].” “I liked that it was easy to implement and was a comparison of similar drugs so some patients were not getting inferior care with this model.” “Chlorthalidone v HCTZ has had disastrous time consuming results, several of these patients wound up in ED for hyponatremia or dehydration. The increased incidence of adverse effects has thus far outweighed benefit of chlorthalidone v hctz. Patients need to be more closely evaluated (both their physiology and their health literacy and behaviors) before trying chlorthalidone.”
Compensation	Providers desire additional compensation when asked to participate in research beyond their normal duties.	“Authorship (not just acknowledgment) in publications [would affect my decision to participate].” “Would prefer to be local PI.” “If you want me to spend time on something, provide compensation for it: reduced panel size, etc. Alternatively, just handle everything within the study: lab f/u, BP f/u, etc. My job as a PCP is not to assist research staff with their projects.”
Importance of Study Question	Provider acceptance of the clinical question to be studied will affect their willingness to participate.	“My perception of the value of the study to the practice of primary care [would affect my decision to participate].“ “I would need to feel the question is important and that our knowledge about the treatments showed them to be close enough in efficacy that it would be ethical to place a patient on either treatment.” “I also wish we had more head to head comparisons of classes of medications, similar to [ALLHAT]. I worry that we are relying so heavily on network meta-analyses for these comparisons.” “Do a research that was not done prior.” “I appreciate the work to understand the difference in these medications. It is a big question in patient care. I am not sure if this helped answer the questions.”
Patient-Provider Relationship	Providers are willing to participate in studies when their relationship and trust with their patients is uncompromised.	“Neg[ative] impact on patient doctor relationship [would affect my decision to participate]” “This study did not affect regular patient care and management did not change as a result of the study. This is was very important to me as a provider. “ “Should not take me away from regular patient care.” “It did not dramatically impact my patient care and patient’s did not feel affected by the options.”

## Discussion

Despite previously reported potential concerns about an increase in clinical burden for providers participating in POC studies, this survey with respect to DCP did not find a meaningful increase, though concern about provider time is evident through the open-ended comments (Table 3**)**. Providers with patients reporting for additional tests or visits because of the study were more likely to describe the trial as burdensome. Though there was no requirement for visits or labs as a result of a medication change, providers may have felt compelled or obligated to schedule additional visits with their patients. For those switching from hydrochlorothiazide to chlorthalidone, it is not unexpected that providers might want to review lab values and add clinical follow-up that was not required by the trial. Thus, for some providers there may be a perceived increase in clinical workload as a result of the trial, though that is highly dependent on a provider’s individual approach to usual care. Interestingly, some respondents could not even recall their participation, potentially indicating the low level of effort required.

Embedded pragmatic trials limit blinding as they are intended to maximize generalizability and typically use objective endpoints that are less prone to bias [16]. Providers and patients being aware of the study medication assignment is not likely to have influenced the cardiovascular outcomes of DCP. However, this may have impacted providers’ responses to the survey questions. Providers who allowed their patients to be randomized to a different diuretic than the one they were taking previously may have felt obligated to conduct lab tests and follow-up with the patient post-randomization, thereby increasing their work burden. Although only a small percentage of consented providers declined randomization of their patients, some providers may have felt disinclined to refuse the randomization because they were concerned that too many randomization declines would affect the ability of the researchers to answer the study question.

The previous focus groups by Weir, et al. [13] indicated the importance of preservation of the provider-patient relationship and their clinical autonomy despite the conduct of a POC trial. In the survey of DCP providers, 99% reported no change or an improvement to their relationship with patients. Only two respondents indicated a negative impact of the trial citing “the provider role was unclear” and “[patients] were a little confused about someone not their PCP making medication adjustments”. DCP was designed to preserve provider autonomy despite participation in the study, and thus there are few comments in the survey indicating concern about this aspect. However, one respondent stated “This study did not affect regular patient care and management did not change as a result of the study. This is/ was very important to me as a provider.” This indicates that this may still be a concern to providers participating in POC trials, and that DCP effectively addressed that concern in its design.

Given the responses about undue burden of the study and comments about the relationship with patients, the importance of education of providers about the trial is evident. Although the DCP study team made significant efforts to ensure awareness of the trial before recruiting providers and patients, the penetration of that information can be improved in future studies. Despite the efforts of the study team, a proportion of providers felt that they had not received enough information prior to their patients enrolling in the trial. Although the providers provided informed consent to their own participation and approved individual patients in DCP, care should be taken to ensure that any participants are adequately informed, whether provider or patient.

Respondents that reported being comfortable with the pragmatic embedded trial design and the study question were also more likely to consider participating in future POC studies. In fact, several respondents indicated a desire to see and be involved in more embedded research in the future. There were also a number of suggestions from respondents on how to improve their desire to participate and improve their experience with these trial designs including potential for authorship, updates or progress reports throughout the study, and increased communication from the study team about the impact of the trial on patients (Table 3).

Our ability to assess the provider experience with DCP by the survey had limitations. Although the survey targeted all providers consented to DCP, only 180 (4%) consented providers completed the survey. The low response rate may have introduced selection bias, though participants and consented providers were roughly the same in measured demographics (Table 1). However, these demographics were limited to age and time in the VA. In some cases, collecting the geographic information from a responding provider may have prevented anonymity, which could then also influence provider responses and introduce response bias. The trial lasted five years and some providers left the VA healthcare system during this time and were not able to complete surveys. Only 13 primary care providers that consented to participate in the study withdrew their participation before the end of the study. The low dropout rate of providers could be an indication of overall satisfaction with the trial design and ease of participation in the study. Additionally, since DCP was conducted in the primary care setting, results of this survey may only be applicable to other providers and trials in primary care. Provider participants in the study had not yet been made aware of the findings of the study at the time the survey was conducted. Consequently, we do not know if provider attitudes towards the trial would have been impacted by learning the results of the study. Provider satisfaction could be affected by the provider’s perception of whether or not the results support changes in clinical practice that will have a positive impact on patient care. The free text option in the survey was meant to capture things that affected the provider experience that were not covered in the survey questions, however, there may have been issues that affected the provider experience that were not captured from the structured questions or the open comments. For future trials, it may be beneficial to query participating providers both before and after the study to better understand providers’ changes in expectations and understanding of these types of trials.

Results of this survey in conjunction with experiences during the course of DCP provide lessons for future pragmatic clinical trials. In particular:

1. Transparent, frequent, and continued communication with providers about their enrolled patients and progress of the trial are highly valued. This includes both information on individual patients as well as aggregate summaries about the trial, when possible. DCP generated annual site-level summaries of recruitment progress for leadership at each of the participating medical centers. In retrospect, additional circulation of these summaries to participating providers would likely have been appreciated.
2. Low participation burden for providers is critical. This can be achieved by aligning study workflows with clinical practice for minimal disruption [17]. DCP was designed to depart minimally from usual primary care and was executed within the EHR environment to which providers are accustomed. This combined with attention to how many study-related requests and orders were sent to providers in a given week minimized the time required and the learning curve experienced by providers.
3. Pragmatic clinical trials represent a paradigm shift in research for many providers, thus education about study design and execution is crucial for high levels of provider participation. Support and understanding from leadership at participating medical centers can go a long way to helping potentially referring and participating providers understand the mechanics and value of pragmatic embedded clinical trials [17].

When designing a POC study, there should be a focus on educating providers and patients and keeping them informed of study progress, minimizing work burden and disruption to usual care practices, and obtaining buy-in from leadership, especially at sites that are less familiar with conducting clinical research.

## Conclusions

In general, responses around experience and participation in the DCP and POC studies were positive and support continued use of embedded trials. Providers found the trial to have limited impact on their clinical duties in line with the intention of the study design and would be willing to be involved in future studies of this design. In the future, provider satisfaction with POC trials may be improved by providing more communication with providers before, during, and after the study.

## Data Availability

The datasets generated and analyzed during the current study are not publicly available but are available on request with an IRB-approved protocol and upon completion of a VA-approved data use agreement.
